# Effectiveness of first and second COVID‐19 mRNA vaccine monovalent booster doses during a period of circulation of Omicron variant sublineages: December 2021–July 2022

**DOI:** 10.1111/irv.13104

**Published:** 2023-03-01

**Authors:** Joshua G. Petrie, Jennifer P. King, David L. McClure, Melissa A. Rolfes, Jennifer K. Meece, David Pattinson, Gabriele Neumann, Yoshihiro Kawaoka, Edward A. Belongia, Huong Q. McLean

**Affiliations:** ^1^ Marshfield Clinic Research Institute Marshfield Wisconsin USA; ^2^ Influenza Division Centers for Disease Control and Prevention Atlanta Georgia USA; ^3^ Department of Pathobiological Sciences, School of Veterinary Medicine University of Wisconsin‐ Madison Wisconsin USA

**Keywords:** booster, COVID‐19, Omicron, SARS‐CoV‐2, vaccine effectiveness, waning

## Abstract

**Background:**

US recommendations for COVID‐19 vaccine boosters have expanded in terms of age groups covered and numbers of doses recommended, whereas evolution of Omicron sublineages raises questions about ongoing vaccine effectiveness.

**Methods:**

We estimated effectiveness of monovalent COVID‐19 mRNA booster vaccination versus two‐dose primary series during a period of Omicron variant virus circulation in a community cohort with active illness surveillance. Hazard ratios comparing SARS‐CoV‐2 infection between booster versus primary series vaccinated individuals were estimated using Cox proportional hazards models with time‐varying booster status. Models were adjusted for age and prior SARS‐CoV‐2 infection. The effectiveness of a second booster among adults ≥50 years of age was similarly estimated.

**Results:**

The analysis included 883 participants ranging in age, from 5 to >90 years. Relative effectiveness was 51% (95% CI: 34%, 64%) favoring the booster compared with primary series vaccination and did not vary by prior infection status. Relative effectiveness was 74% (95% CI: 57%, 84%) at 15 to 90 days after booster receipt, but declined to 42% (95% CI: 16%, 61%) after 91 to 180 days, and to 36% (95% CI: 3%, 58%) after 180 days. The relative effectiveness of a second booster compared to a single booster was 24% (95% CI: −40% to 61%).

**Conclusions:**

An mRNA vaccine booster dose added significant protection against SARS‐CoV‐2 infection, but protection decreased over time. A second booster did not add significant protection for adults ≥50 years of age. Uptake of recommended bivalent boosters should be encouraged to increase protection against Omicron BA.4/BA.5 sublineages.

## 1 INTRODUCTION

Clinical trials supporting emergency use authorization of COVID‐19 messenger RNA (mRNA) vaccines in the United States estimated the efficacy of a two‐dose series to be >90% in preventing symptomatic infection.^1^, ^2^ Post‐authorization observational studies continued to estimate high vaccine effectiveness (VE) against symptomatic infection, even after the emergence of the SARS‐CoV‐2 Alpha and Delta variants.^3^ In November 2021, a monovalent booster dose of COVID‐19 vaccine targeting the original SARS‐CoV‐2 strain was recommended for high‐risk groups, citing concerns of waning immunity, decreasing VE, and increasing Delta variant incidence throughout the United States.^4^ Recommendations for a booster dose were gradually expanded to include all people aged ≥12 years in the United States as of January 5, 2022 in an effort to improve protection against the Omicron variant, which was rapidly increasing in the United States.^5^

In contrast to previous SARS‐CoV‐2 variants of concern, the Omicron variant evaded immunity from prior infection and incidence of breakthrough infections among the vaccinated increased.^6^, ^7^ Yet, VE against severe outcomes remained high, particularly among those who received a booster dose.^8^ Subsequent studies have also demonstrated the effectiveness of a booster dose in preventing symptomatic Omicron infection, though protection declined with time since vaccination.^9^, ^10^, ^11^, ^12^, ^13^, ^14^ However, most of these studies identified infections in healthcare settings where testing and healthcare seeking behaviors may impact results.

Omicron variants have since diversified into a number of sublineages including BA.2, BA.4, and BA.5. These sublineages, particularly BA.4/BA.5, exhibit reduced neutralization by antibodies induced by vaccination and infection with the original Omicron BA.1 sublineage.^15^, ^16^, ^17^ A limited number of studies of the effectiveness of COVID‐19 vaccine booster doses against BA.4/BA.5 infection have been published suggesting continued benefit against severe infection.^18^, ^19^ Vaccination recommendations in the United States have also continued to evolve. On March 29, 2022, a second monovalent booster dose targeting the original SARS‐CoV‐2 was authorized and recommended for US adults aged ≥50 years and individuals with immunocompromising conditions.^20^ Children of ages five to 11 were then authorized to receive a first booster dose on May 17, 2022.^21^

During a period of circulation of Omicron variant sublineages, we estimated relative VE of mRNA booster vaccination versus two‐dose primary series in an ongoing community cohort with active illness surveillance in rural central Wisconsin, USA. We also estimated the relative VE of a second booster versus a single booster in adults aged ≥50 years.

## 2 METHODS

### 2.1 Study population and data collection

The Prospective Assessment of COVID‐19 in a Community (PACC) study is an ongoing longitudinal cohort. Participants in the overall PACC cohort include persons of any age who were randomly sampled and recruited from a defined community cohort in which nearly all residents receive care from the Marshfield Clinic Health System (MCHS).^22^ Participant characteristics including age, sex, and presence of chronic health conditions (Table S1) were determined by survey at enrollment. COVID‐19 vaccination status was determined from MCHS electronic health records, the Wisconsin Immunization Registry, and participant vaccination cards. Blood was collected for serologic studies at enrollment (November 2020–March 2021), and at scheduled visits approximately 12 (January–June 2021) and 24 weeks (April–September 2021) after enrollment. A fourth round of serum collection began in February 2022 (February–May), with a fifth specimen collected approximately 12 weeks later (May–July 2022).

This study was reviewed and approved by the Institutional Review Board at the Marshfield Clinic Research Institute. Participants, or parents of minor participants, provided informed consent prior to participation. Participating children ≥7 years also provided assent for participation.

### 2.2 SARS‐CoV‐2 identification

Participants completed a weekly symptom survey and were instructed to immediately report the onset of new respiratory symptoms. During the entire study period, an anterior nasal swab was self‐ or parent‐collected when participants reported ≥1 of the following symptoms: fever, cough, loss of smell or taste, sore throat, muscle/body aches, shortness of breath, or diarrhea. In addition, participants self‐ or parent‐collected a specimen each week, regardless of symptoms, during the period of January 27, 2022 to July 28, 2022. Compliance with weekly swabbing was over 85%. Study specimens were tested by reverse transcription polymerase chain reaction (RT‐PCR) for identification of SARS‐CoV‐2. SARS‐CoV‐2 infection was additionally defined by a positive molecular clinical test result documented in the MCHS electronic health record, participant self‐report of a positive SARS‐CoV‐2 test via periodic survey, or serologic evidence of infection. Serologic evidence of infection was specifically defined as being seropositive for SARS‐CoV‐2 spike or spike receptor domain antibody among unvaccinated participants, or nucleocapsid protein binding antibody seropositive regardless of vaccination status, as measured by ELISA. Seropositivity thresholds were derived from logistic regression on ELISA measurements against pre‐pandemic and RT‐PCR positive samples (see Pattinson *et al* ^23^ for details). SARS‐CoV‐2 variant proportions in Wisconsin over time were determined using GISAID data compiled at covariants.org.^24^, ^25^

### 2.3 Statistical analysis

The primary analysis estimated the effectiveness of a monovalent COVID‐19 mRNA vaccine booster dose relative to completion of the two‐dose primary series. This analysis included vaccinated PACC participants followed from December 20, 2021 to June 28, 2022 who were aged ≥5 years and were booster‐dose eligible. Booster‐dose eligibility was based on age‐specific dates of booster authorization and being ≥5 months post completion of the mRNA vaccine primary series. Individuals who received Ad26.COV2.S (Johnson & Johnson [Janssen]) (*N* = 46) or mixed mRNA products (*N* = 1) for their primary series were excluded. Participants were described according to age groups corresponding to those for which COVID‐19 vaccine authorization, recommendations, and prioritization have been based (5–11, 12–17, 18–49, 50, and ≥65 years of age). Sparse data required collapsing some of these age groups (e.g., 5–17 and ≥50 years of age) for age stratified VE estimates.

Hazard ratios comparing SARS‐CoV‐2 infection among booster versus primary series vaccinated individuals were estimated using Cox proportional hazards models with time‐varying booster status. The primary outcome modeled was SARS‐CoV‐2 identified by research or clinical testing. Primary series vaccinated person‐time began on December 20, 2021 or upon booster eligibility, whichever was later, and ended upon infection, receipt of the booster dose, end of study period, or withdrawal. Booster dose vaccinated person‐time began on December 20, 2021 or ≥14 days after receipt of the booster dose, whichever was later, and ended upon infection, receipt of a fourth dose, end of study period, or withdrawal. Relative VE of booster versus primary series vaccination was calculated as 100*(one minus the adjusted hazard ratio). Models were adjusted for age (cubic spline with five knots) a priori; sex, presence of chronic health conditions, and prior SARS‐CoV‐2 infection were evaluated as potential confounders. Prior SARS‐CoV‐2 infection was defined as a positive molecular SARS‐CoV‐2 test result from research or clinical testing, self‐report of a positive SARS‐CoV‐2 test via periodic survey, or serologic evidence of infection (Table S2). Only prior SARS‐CoV‐2 infection was retained with age in final models as sex and chronic conditions did not change the effect estimated by ≥10%.

We hypothesized that immunocompromised state or having previous COVID‐19 infection might modify VE. Therefore, we performed the following sensitivity analyses to estimate relative VE: (1) excluding the small number of participants who reported that they were in an immunocompromised state and (2) stratified by evidence of SARS‐CoV‐2 infection prior to person‐time entry.

Waning effectiveness of the booster dose of COVID‐19 mRNA vaccine relative to completion of the 2 dose primary series was also assessed using Cox proportional hazards models adjusted for age and prior SARS‐CoV‐2 infection. Time‐varying vaccination status was categorized as two‐dose primary series (referent), booster dose received 0 to 14 days ago, booster dose received 15 to 90 days ago, booster dose received 91 to 180 days ago, and booster dose received >180 days ago. Relative VE of each dose‐time category was calculated as 100*(one minus the adjusted hazard ratio), completion of two dose primary series as the referent category.

The effectiveness of a second monovalent COVID‐19 mRNA vaccine booster dose relative to receipt of a single booster dose was estimated among PACC participants ≥50 years of age who were followed from March 29, 2022 to July 28, 2022 and were eligible for a fourth dose ≥4 months post booster dose receipt. Statistical methods were similar to those for the analysis comparing single booster dose effectiveness relative to primary series.

## 3 RESULTS

**FIGURE 1:**
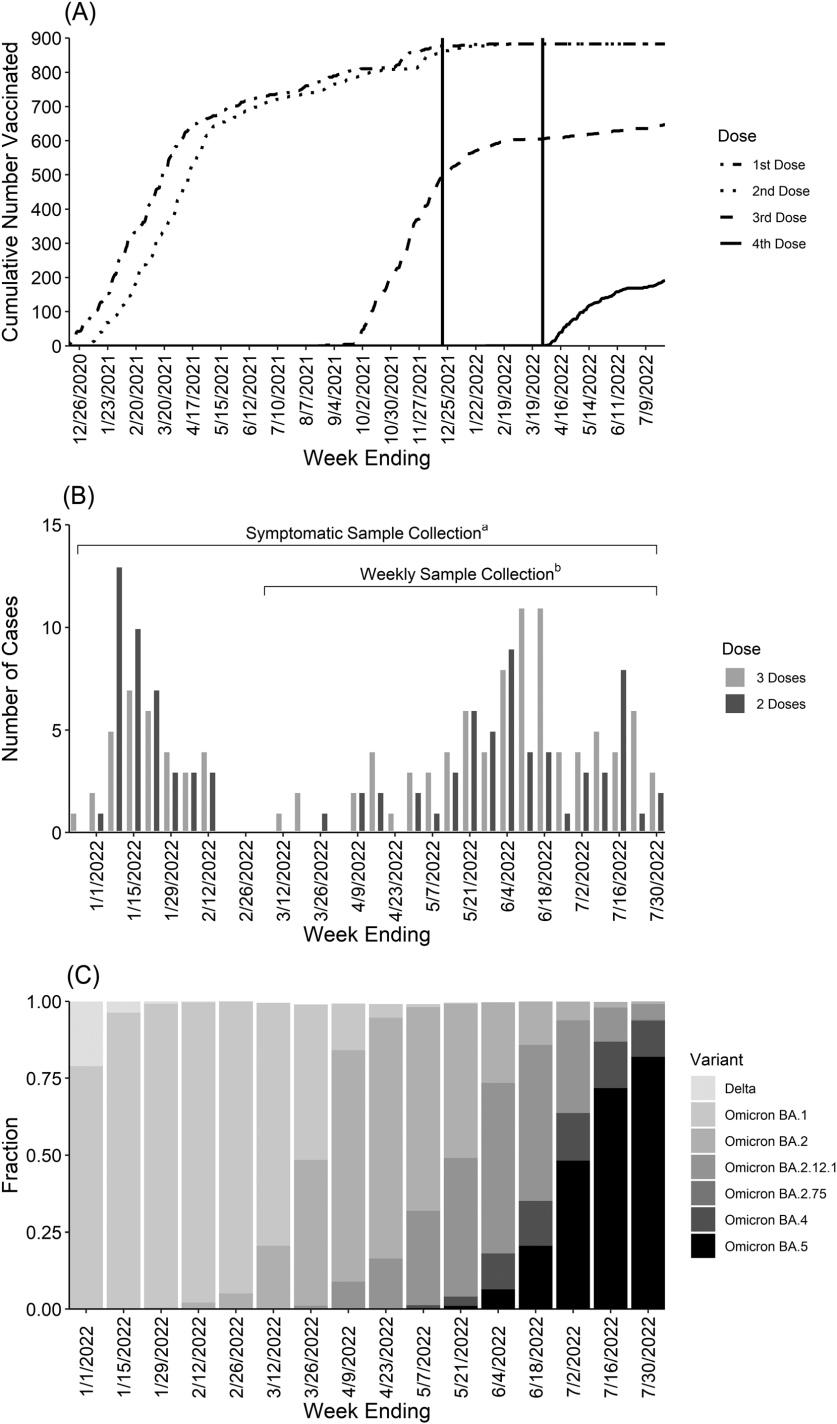
Timing of vaccination and infection Prospective Assessment of COVID‐19 in a Community (PACC) study December 20, 2021–July 28, 2022. (A) Cumulative uptake of mRNA vaccine doses by week. Vertical lines at December 20, 2021 and March 29, 2022 represent the start of the booster and second booster analysis periods, respectively. (B) Weekly RT‐PCR‐confirmed SARS‐CoV‐2 infections by booster status. (C) Variant proportions in Wisconsin over time. ^a^ Self‐collection of nasal swab during illness with ≥1 qualifying symptom (fever, cough, loss of taste/smell, sore throat, muscle/body aches, shortness of breath, or diarrhea). ^b^ Self‐collection of nasal swab every week, regardless of symptoms.

There were 883 participants included in the analysis. Participants ranged in age from 5 to >90 years (median: 55, IQR: 34–71), 61% were female, and 56% had ≥1 chronic health condition (Table 1). A total of 258 (29%) participants completed the primary series without boosting, and 625 (71%) received a booster dose by the end of follow‐up (Figure 1A). A total of 102,842 person‐days were contributed following booster dose of COVID‐19 vaccine, and 35,034 person‐days were contributed by unboosted individuals. The median time from completion of the primary series to the start of follow‐up was 169 days (range: 152 to 347) for unboosted participants and 274 days (range: 152 to 350) for boosted participants. The median time from booster dose to the start of follow‐up was 32 days (range: 0 to 125) for boosted participants; 22% of boosted individuals received their booster dose during follow‐up and were considered to have 0 day between booster dose receipt to the start of follow‐up. Among unboosted individuals, 205 (79%) received two doses of BTN162b2 (Pfizer‐BioNTech Comirnaty) vaccine, and 53 (21%) received two doses of mRNA‐1273 (Moderna Spikevax) vaccine. Among boosted individuals, 384 (61%) received two doses of BTN162b2, 241 (39%) received two doses of mRNA‐1273. Booster doses were 65% BTN162b2 and 35% mRNA‐1273.

**TABLE 1:** Characteristics of participants included in analysis of relative effectiveness of a booster dose of COVID‐19 vaccination versus two‐dose primary series by booster and infection status: Prospective Assessment of COVID‐19 in a Community (PACC) study December 20, 2021–July 28, 2022.

	Booster vaccinated^a^			Primary series vaccinated ≥5 months^b^		
	SARS‐CoV‐2 Infected^c^ no. (%)	SARS‐CoV‐2 Uninfected^c^ no. (%)	Total no. (%)	SARS‐CoV‐2 Infected^c^ no. (%)	SARS‐CoV‐2 Uninfected^c^ no. (%)	Total no. (%)
Age Group (years)						
5–11	1 (0.8)	9 (1.8)	10 (1.6)	17 (17.5)	34 (21.1)	51 (19.8)
12–17	5 (4.2)	24 (4.7)	29 (4.6)	16 (16.5)	30 (18.6)	46 (17.8)
18–49	47 (39.8)	121 (23.9)	168 (26.9)	41 (42.3)	49 (30.4)	90 (34.9)
50–64	31 (26.3)	105 (20.7)	136 (21.8)	17 (17.5)	36 (22.4)	53 (20.5)
≥65	34 (28.8)	248 (48.9)	282 (45.1)	6 (6.2)	12 (7.5)	18 (7.0)
Sex						
Female	69 (58.5)	312 (61.5)	381 (61.0)	65 (67.0)	89 (55.3)	154 (59.7)
Male	49 (41.5)	195 (38.5)	244 (39.0)	32 (33.0)	72 (44.7)	104 (40.3)
Chronic Condition(s)^d^						
Yes	61 (51.7)	326 (64.3)	387 (61.9)	35 (36.1)	69 (42.9)	104 (40.3)
No	57 (48.3)	181 (35.7)	238 (38.1)	62 (63.9)	92 (57.1)	154 (59.7)
Immune Compromised^e^						
Yes	8 (6.8)	39 (7.7)	47 (7.5)	4 (4.1)	5 (3.1)	9 (3.5)
No	110 (93.2)	468 (92.3)	578 (92.5)	93 (95.9)	156 (96.9)	249 (96.5)
Prior SARS‐CoV‐2 Infection^f^						
Yes	20 (16.9)	174 (34.3)	194 (31.0)	33 (34.0)	97 (60.2)	130 (50.4)
No	98 (83.1)	333 (65.7)	431 (69.0)	64 (66.0)	64 (39.8)	128 (49.6)

^a^ Receipt of three doses of SARS‐CoV‐2 messenger RNA vaccine. Individuals are considered boosted ≥14 days after receipt of their third dose.

^b^ Receipt of two doses of SARS‐CoV‐2 messenger RNA vaccine and eligible for booster dose.

^c^ SARS‐CoV‐2 infection status during the follow‐up period December 20, 2021–July 28, 2022.

^d^ At enrollment, participants were asked to report any of the following chronic conditions: asthma, cancer, chronic kidney disease, COPD, hypertension, immunocompromised state, serious heart condition (heart failure, coronary artery disease or cardiomyopathies), or Type 2 diabetes.

^e^ Immune compromised state self‐reported by study participant at enrollment.

^f^ SARS‐CoV‐2 infection prior to December 20, 2021 or person‐time entry, whichever is later.

A total of 219 (25%) SARS‐CoV‐2 infections were identified, 97 (27.7/10000 person‐days) occurred after receiving the primary series, 122 after a booster dose (11.5/10000 person‐days) (Figure 1B). Of the 122 post‐booster infections, four occurred in the first 14 days after vaccination and were excluded from primary analysis, but were retained for analyses considering time since vaccination. Among the 219 infected individuals, 11 had a second infection identified after their person‐time contribution ended. The proportion of SARS‐CoV‐2 viruses detected in Wisconsin that were identified as Omicron variant increased rapidly in December 2021, accounting for over 75% of the viruses detected in the last 2 weeks of the year (Figure 1C). Omicron variant sublineages accounted for nearly 100% of detected viruses for the remainder of the study period with BA.1 viruses predominating from December 2021 to March 2022, BA.2 viruses from April to June 2022, and BA.4 and BA.5 viruses during July 2022.

**FIGURE 2:**
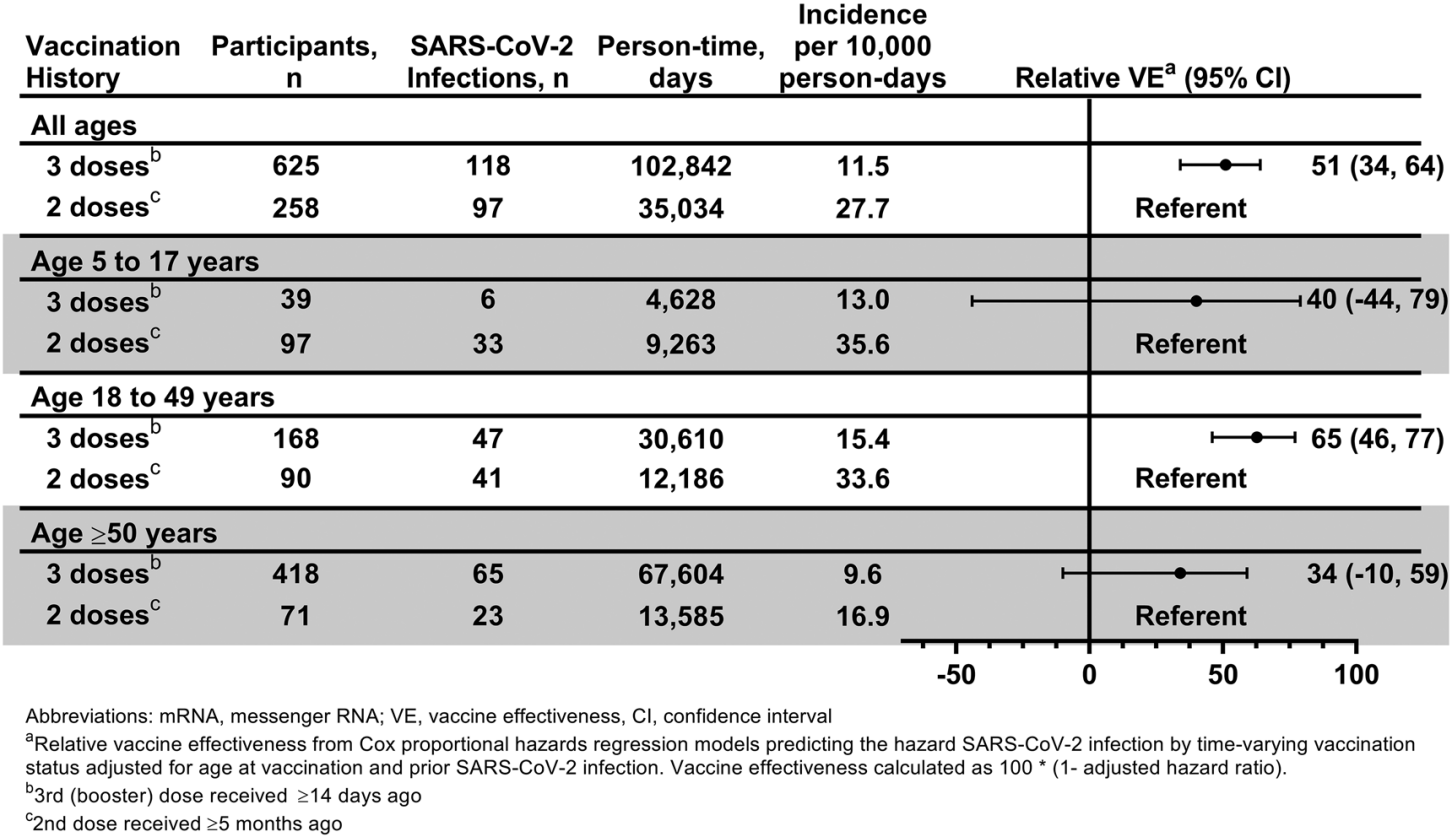
Effectiveness of a booster dose of COVID‐19 mRNA vaccine against laboratory‐confirmed symptomatic SARS‐CoV‐2 infection relative to a two‐dose primary series of COVID‐19 mRNA vaccine: Prospective Assessment of COVID‐19 in a Community (PACC) study December 20, 2021–July 28, 2022. Abbreviations: mRNA, messenger RNA; VE, vaccine effectiveness. ^a^Relative vaccine effectiveness from Cox proportional hazards regression models predicting the hazard SARS‐CoV‐2 infection by time‐varying vaccination status adjusted for age and evidence of SARS‐CoV‐2 infection prior to person‐time entry. Vaccine effectiveness calculated as 100* (one minus the adjusted hazard ratio). ^b^Booster dose received ≥14 days ago. ^c^Second dose received ≥5 months ago.

Relative VE was estimated to be 51% (95% CI: 34%, 64%) favoring the booster dose compared with primary series completion only (Figure 2). Relative VE did not significantly vary by age group (5 to 17, 18 to 49, and ≥50 years), but point estimates were lower in the 5 to 17 and ≥50 year age groups and only the estimate for the 18 to 49 year age group was statistically significant 65% (95% CI: 46%, 77%). SARS‐CoV‐2 infection prior to person‐time entry was identified in 194 (31%) boosted participants and 130 (50%) primary series participants. Reinfection was common during the analysis period, including 20 of 194 (10%) boosted participants and 33 of 130 (25%) primary series participants with prior infection. Relative VE was similar when stratifying by prior infection status at 56% (95% CI: 17%, 77%) among those with evidence of prior infection and 51% (95% CI: 31%, 65%) among those without. Relative VE did not change when excluding 54 participants with an immunocompromising condition defined by self/guardian report (51% [95% CI: 33%, 64%]).

**FIGURE 3:**
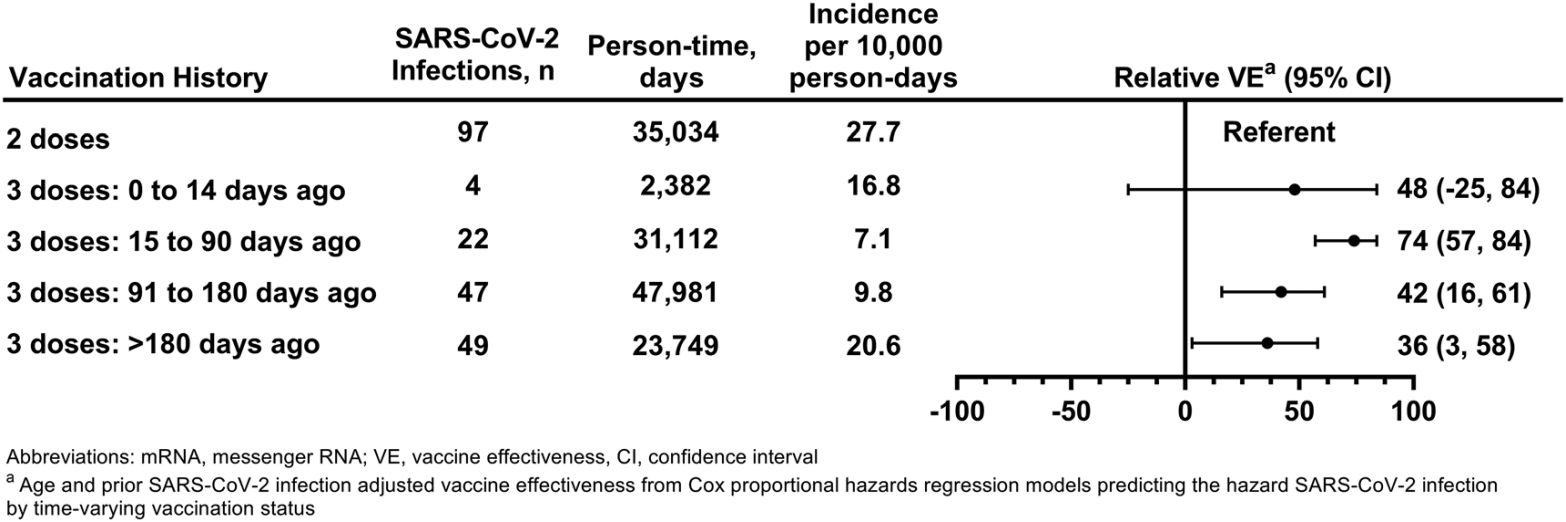
Effectiveness of a booster dose of COVID‐19 mRNA vaccine against laboratory‐confirmed symptomatic SARS‐CoV‐2 infection relative to a two‐dose primary series of COVID‐19 mRNA vaccine by time from last vaccine dose receipt: Prospective Assessment of COVID‐19 in a Community (PACC) study December 20, 2021–July 28, 2022. Abbreviations: mRNA, messenger RNA; VE, vaccine effectiveness. ^a^Relative vaccine effectiveness from Cox proportional hazards regression models predicting the hazard SARS‐CoV‐2 infection by time‐varying vaccination status adjusted for age and evidence of SARS‐CoV‐2 infection prior to person‐time entry.

We observed decreasing relative effectiveness of the booster dose versus primary series vaccination with increasing time from booster receipt (Figure 3). Compared with individuals who had only completed a two‐dose primary series, relative VE was highest in the period of 15 to 90 days after booster receipt (74% [95% CI: 57%, 84%]). Relative VE decreased to 42% (95% CI: 16%, 61%) in the period of 91 to 180 days after booster receipt, and to 36% (95% CI: 3%, 58%) after 180 days.

**FIGURE 4:**
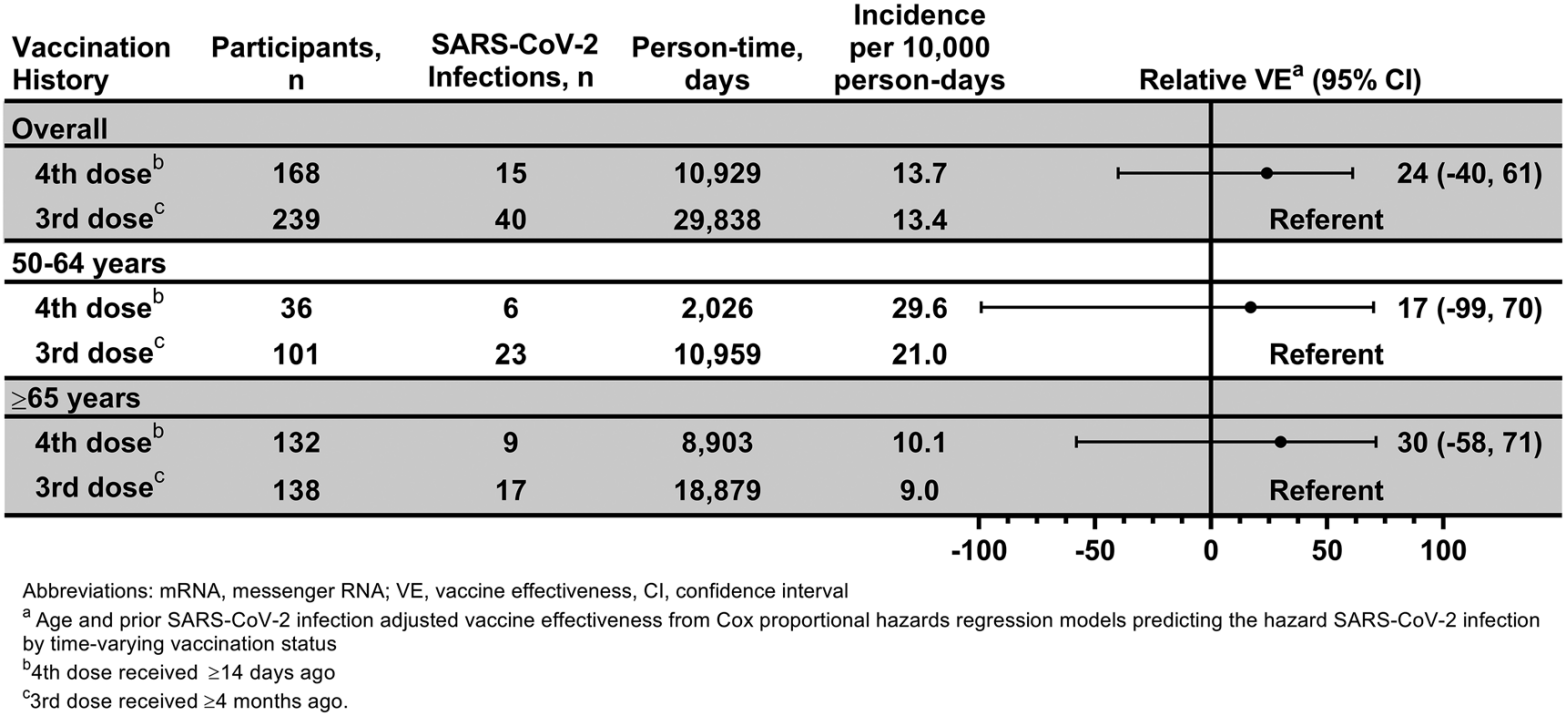
Effectiveness of a second booster dose of COVID‐19 mRNA vaccine against laboratory‐confirmed symptomatic SARS‐CoV‐2 infection relative to single booster receipt among adults ≥50 years of age: Prospective Assessment of COVID‐19 in a Community (PACC) study March 29, 2022–July 28, 2022. Abbreviations: mRNA, messenger RNA; VE, vaccine effectiveness. ^a^Relative vaccine effectiveness from Cox proportional hazards regression models predicting the hazard SARS‐CoV‐2 infection by time‐varying vaccination status adjusted for age and evidence of SARS‐CoV‐2 infection prior to person‐time entry. Vaccine effectiveness calculated as 100* (one minus the adjusted hazard ratio). ^b^Second booster (4th) dose received ≥14 days ago. ^c^First booster (3rd) dose received ≥4 months ago.

The effectiveness of a second booster of COVID‐19 mRNA vaccine was assessed relative to receipt of a single booster dose among 407 participants ≥50 years of age. Among this subset, 57% were female, and 71% had ≥1 chronic health condition, 7% reported that they were immune compromised, and 38% had evidence of SARS‐CoV‐2 infection prior to person‐time entry (Table 2). A second booster dose was received by 168 (41%), and 239 (59%) received only a single booster. Risk of SARS‐CoV‐2 infection did not significantly vary among single and second booster dose recipients overall, or within the 50–64 and ≥65 year age groups (Figure 4).

**TABLE 2:** Characteristics of participants ≥50 years of age included in analysis of the effectiveness of a second booster dose of COVID‐19 mRNA vaccine relative to single booster recipients by booster and infection status: Prospective Assessment of COVID‐19 in a Community (PACC) study March 29, 2022–July 28, 2022.

	Second booster vaccinated (four doses)^a^			Booster vaccinated (three doses)^b^		
	SARS‐CoV‐2 Infected^c^ no. (%)	SARS‐CoV‐2 Uninfected^c^ no. (%)	Total no. (%)	SARS‐CoV‐2 Infected^c^ no. (%)	SARS‐CoV‐2 Uninfected^c^ no. (%)	Total no. (%)
Age Group (years)						
50–64	6 (40.0)	30 (19.6)	36 (21.4)	23 (57.5)	78 (39.2)	101 (42.3)
≥65	9 (60.0)	123 (80.4)	132 (78.6)	17 (42.5)	121 (60.8)	138 (57.7)
Sex						
Female	9 (60.0)	87 (56.9)	96 (57.1)	19 (47.5)	119 (59.8)	138 (57.7)
Male	6 (40.0)	66 (43.1)	72 (42.9)	21 (52.5)	80 (40.2)	101 (42.3)
Chronic Condition(s)^d^						
Yes	10 (66.7)	116 (75.8)	126 (75.0)	23 (57.5)	139 (69.8)	162 (67.8)
No	5 (33.3)	37 (24.2)	42 (25.0)	17 (42.5)	60 (30.2)	77 (32.2)
Immune Compromised^e^						
Yes	3 (20.0)	9 (5.9)	12 (7.1)	2 (5.0)	15 (7.5)	17 (7.1)
No	12 (80.0)	144 (94.1)	156 (92.9)	38 (95.0)	184 (92.5)	222 (92.9)
Prior SARS‐CoV‐2 Infection^f^						
Yes	0 (0.0)	52 (34.0)	52 (31.0)	14 (35.0)	90 (45.2)	104 (43.5)
No	15 (100)	101 (66.0)	116 (69.0)	26 (65.0)	109 (54.8)	135 (56.5)

^a^ Receipt of four doses of SARS‐CoV‐2 messenger RNA vaccine. Individuals are considered twice boosted ≥14 days after receipt of their fourth dose.

^b^ Receipt of three doses of SARS‐CoV‐2 messenger RNA vaccine and eligible for a second booster dose.

^c^ SARS‐CoV‐2 infection status during the follow‐up period March 29, 2022–July 28, 2022.

^d^ At enrollment, participants were asked to report any of the following chronic conditions: asthma, cancer, chronic kidney disease, COPD, hypertension, immunocompromised state, serious heart condition (heart failure, coronary artery disease or cardiomyopathies), or Type 2 diabetes.

^e^ Immune compromised state self‐reported by study participant at enrollment.

^f^ SARS‐CoV‐2 infection prior to December 20, 2021 or person‐time entry, whichever is later.

## 4 DISCUSSION

In our study, a monovalent mRNA vaccine booster dose targeting ancestral SARS‐CoV‐2 reduced the risk of SARS‐CoV‐2 infection by 51% during a period of circulation of Omicron and Omicron variant sublineages. These findings are consistent with estimates of booster effectiveness against symptomatic Omicron infection using healthcare ascertainment.^9^, ^10^, ^11^, ^12^, ^13^, ^14^ As of October 2022, 68% of the US population has completed primary series vaccination.^26^ Among those fully vaccinated individuals ≥5 years of age who are booster eligible, only 49% have received a booster dose. It is clear that COVID‐19 vaccine booster doses have been very effective in preventing hospitalization and death,^8^, ^10^, ^13^ but uncertainty around effectiveness in preventing infection may contribute to vaccine hesitancy in some populations. Other than concerns of adverse effects, concerns over effectiveness were the most commonly reported reasons for COVID‐19 vaccine hesitancy in a large US cohort.^27^ Our study provides important evidence that booster doses were effective in preventing Omicron infection in the community among individuals with and without prior infection.

It has been demonstrated that serum from recently boosted individuals has greatly improved capacity to neutralize Omicron than serum from individuals who recently completed two‐dose primary series vaccination suggesting increased breadth of response.^28^ However, boosting immunity that waned following primary series vaccination likely also contributes to booster dose protection. This latter mechanism is consistent with our finding of decreasing protection with time since receipt of the booster dose. Waning effectiveness of the booster dose has also been demonstrated in a number of other studies with protection against infection decreasing more sharply than protection against severe infection.^9^, ^13^, ^29^, ^30^, ^31^

We found little variation in the relative effectiveness of mRNA booster doses by age; however, relative effectiveness was only statistically significant among the 18 to 49 year age group, potentially reflecting small sample sizes in other age groups. Although not statistically significant, our results suggest that booster doses did reduce the risk of Omicron infection among adolescents when interpreted in the context of other recent studies. Specifically, our relative VE point estimate of 40% (95% CI: −44%, 79%) in the 5 to 17 year age group is consistent with other, larger studies of the effectiveness of boosters in adolescents.^32^, ^33^

In our study, a second booster did not provide significant added protection against infection over a first booster for adults ≥50 years of age during a period of primarily Omicron BA.2 and BA.5 sublineage circulation. A second BTN162b2 booster was previously found to be 52% and 72% effective in preventing Omicron BA.1 infection and hospitalization, respectively, in adults ≥60 years.^34^ Subsequent studies that were performed in periods when a mix of BA.1, BA.2, BA.4, and BA.5 viruses circulated have reported low effectiveness of a second booster against infection, but higher effectiveness against more severe disease.^18^, ^19^, ^35^, ^36^ Waning protection after monovalent boosters and the emergence of antigenically distinct sublineages support the need for bivalent mRNA boosters containing spike protein mRNA of both the original SARS‐CoV‐2 virus and Omicron variant, which are now available in the United States and other countries.

Strengths of our study include the prospective cohort design with broad age representation, well‐characterized infection history, and active surveillance for SARS‐CoV‐2 infection outside of the healthcare setting. However, our results should be interpreted in the context of a number of limitations. First, although we adjusted for confounding by age and prior SARS‐CoV‐2 infection, our results may be subject to residual confounding due to unmeasured factors as in any observational study. For example, we were unable to account for time since the last prior infection, and there could have been misclassification of self‐reported chronic health conditions. Second, sample sizes were limited particularly for subgroup analyses by age, and we likely missed asymptomatic BA.1 infections that occurred before weekly swabbing started. Third, although we detected decreasing relative effectiveness over time, we are unable to disentangle the effects of waning immunity and antigenic changes in the circulating viruses over time. Finally, interpretation of relative VE is not straightforward and depends on the absolute VE in the referent group.

VE against symptomatic SARS‐CoV‐2 infection in the community provides additional evidence that booster recommendations were effective. However, a second monovalent booster did not provide significant added protection against SARS‐CoV‐2 infection for older adults. Our results also further highlight the fact that vaccine‐induced protection against SARS‐CoV‐2 infection is temporary and is impacted by waning immunity as well as evolution of circulating virus. Increasing bivalent booster uptake should be a priority to increase protection lost likely lost due to waning immunity and antigenic change of circulating viruses. As is the case with influenza, routine vaccination, and regular evaluation of vaccine composition will be necessary to address ongoing SARS‐CoV‐2 transmission.

## AUTHOR CONTRIBUTIONS

**Joshua G. Petrie**: Conceptualization; formal analysis; methodology; writing original draft. **Jennifer P. King**: Conceptualization; methodology; project administration. **David L. McClure**: Conceptualization; formal analysis; methodology. **Melissa A. Rolfes**: Conceptualization; investigation; methodology; project administration; supervision. **Jennifer K. Meece**: Conceptualization; methodology; project administration; supervision. **David Pattinson**: Conceptualization; data curation; methodology. **Gabriele Neumann**: Conceptualization; methodology; project administration; supervision. **Yoshihiro Kawaoka**: Conceptualization; funding acquisition; methodology; supervision. **Edward A. Belongia**: Conceptualization; methodology; supervision. **Huong Q. McLean**: Conceptualization; funding acquisition; project administration; methodology; supervision.

## CONFLICT OF INTEREST STATEMENT

HQM and DLM report research support from Seqirus outside the submitted work. JKM reports research support from Quidel and Seqirus outside the submitted work. All other authors report no potential conflicts.

## ETHICS APPROVAL STATEMENT

This study was reviewed and approved by the Institutional Review Board at the Marshfield Clinic Research Institute.

### PEER REVIEW

The peer review history for this article is available at https://publons.com/publon/10.1111/irv.13104.

## DISCLAIMER

The findings and conclusions in this report are those of the author(s) and do not necessarily represent the official position of the Centers for Disease Control and Prevention (CDC).

## PATIENT CONSENT STATEMENT

Participants, or parents of minor participants, provided informed consent prior to participation. Participating children ≥7 years also provided assent for participation.

## Supporting information


**Table S1.** Chronic health conditions PACC participants were asked to report during study interviews.
**Table S2.** Method of identification of SARS‐CoV‐2 infections occurring prior to person‐time analysis entry.

## Data Availability

The data that support the findings of this study are available upon reasonable request from the corresponding author. The data are not publicly available due to privacy or ethical restrictions.
