# Supplementary material for: Effectiveness of first and second COVID‐19 mRNA vaccine monovalent booster doses during a period of circulation of Omicron variant sublineages: December 2021–July 2022
Source: Influenza Other Respir Viruses. 2023 Mar 1;17(3):e13104. doi: 10.1111/irv.13104 (PMC9975792; doi:10.1111/irv.13104)
Supplement: Supplementary file 1 — Table S1. Chronic health conditions PACC participants were asked to report during study interviews. Table S2. Method of identification of SARS‐CoV‐2 infections occurring prior to person‐time analysis entry. [file IRV-17-e13104-s001.docx]

**Supplemental Table 1.** Chronic health conditions PACC participants were asked to report during study interviews.

| **[Do you/Does your child] currently have any of the following health conditions requiring medical care?** |
| --- |
| Asthma  Cancer |
| Chronic Kidney Disease |
| Chronic Obstructive Pulmonary Disease |
| Other Chronic Lung Disease |
| Hypertension |
| Immunocompromised State |
| Serious Heart Condition (Heart Failure, Coronary Artery Disease, or Cardiomyopathy) |
| Type 1 Diabetes |
| Type 2 Diabetes |
| Chronic Liver Disease |
| Mental Health Conditions |

**Supplemental Table 2.** Method of identification of SARS-CoV-2 infections occurring prior to person-time analysis entry.

|  | No. (%) |
| --- | --- |
| Molecular test only | 17 (6) |
| Self-report only | 13 (4) |
| Serology only | 130 (37) |
| Molecular & self-report | 25 (7) |
| Molecular & serology | 34 (12) |
| Self-report & serology | 16 (5) |
| Molecular, self-report, & serology | 89 (29) |
